# Traumatic fracture of central venous catheter

**DOI:** 10.21542/gcsp.2024.16

**Published:** 2024-03-03

**Authors:** Feridoun Sabzi, Reza Faraji, Arezoo Bozorgomid

**Affiliations:** 1 Department of General Surgery, School of Medicine, Kermanshah University of Medical Sciences, Kermanshah, Iran; 2 Tuberculosis and Lung Diseases Research Center, Ilam University of Medical Sciences, Ilam, Iran; 3 Infectious Diseases Research Center, Health Institute, Kermanshah University of Medical Sciences, Kermanshah, Iran; 4 Department of Medical Parasitology and Mycology, School of Medicine, Kermanshah University of Medical Sciences, Kermanshah, Iran

## Abstract

Central venous catheter (CVC) placement is frequently essential for the management of critically ill patients. This report describes a case involving the surgical retrieval of an embolized fragment of a CVC, originally intended for dialysis, following an unsuccessful removal attempt by a nurse due to catheter malfunction. The decision to remove and replace the malfunctioning catheter was made by the patient’s physician. However, during the removal process, both the retaining suture and the catheter were inadvertently severed. This report also discusses the complications and management strategies associated with the embolization of a central line.

## Introduction

Central venous catheters (CVC) are used commonly in renal failure for hemodialysis. Cases of dialysis catheter tip thrombosis that iatrogenically ruptured and embolized to the right atrium are very rare. When the catheter tip thrombus is present, it carries a mortality risk of 18% in hemodialysis patients and greater than 40% risk in others patients^1^.

Different pathogenic mechanisms have been postulated for the development of CVC thrombosis, including mechanical irritation of the myocardial wall, propagation of intraluminal clot, hyper coagulopathy, and hemodynamic of right atrium^2^. Presentation of CVC thrombosis may be asymptomatic or with an associated complication such as pulmonary embolism, systemic embolism, infected thrombi, or hemodynamic compromise^3^. We describe an interesting case of a 59-year-old asymptomatic male, successfully treated with beating heart surgery after migration of an inadvertent fracture of catheter tip in his right atrium. Our case underscores that beating heart surgery of CVC thrombosis may carry a favorable prognosis as opposed to open heart surgery that may increased catastrophic complications of cardiopulmonary bypass.

### Case presentation

A 59-year-old male patient with significant morbidity, including hemodialysis-dependent chronic renal failure, coronary artery disease, atrial fibrillation, chronic obstructive pulmonary disease, and cirrhosis of the liver. He was admitted to the our heart center with an embolized split catheter to the right atrium following inadvertent tearing of catheter by nurses.

While the patient was in the ward, a malfunctioning catheter prompted the nephrologist to remove it from the right atrium. During the retrieval process, the catheter was accidentally cut. Consequently, the thrombosis weight at the catheter tip caused it to be drawn from the right internal jugular vein into the right atrium. Following thorough investigations and stabilization of the patient’s general condition, a median sternotomy procedure was planned and carried out under general anesthesia.

**Figure 1.**
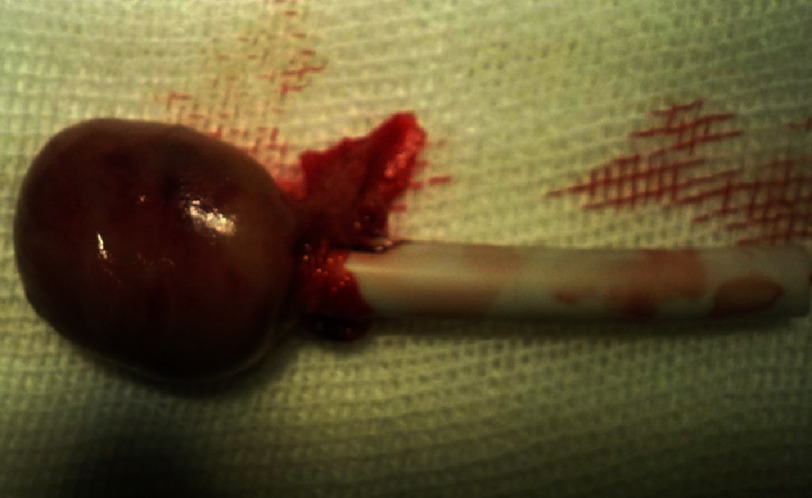
Large catheter tip thrombosis.

Surgery revealed that the tip of the fractured catheter was free in right atrium and was not attached to the atrial wall or tricuspid wall. The catheter tip was retained by two fingers in wall of right atrium and with small right atriotomy incision, it was retrieved. On examination of the cut end of the catheter, there was a large organized thrombosis that attached to catheter tip (Figure 1). We think that the weight of the thrombosis in the catheter tip caused a rapid fall of the catheter to right atrium.

**Figure 2.**
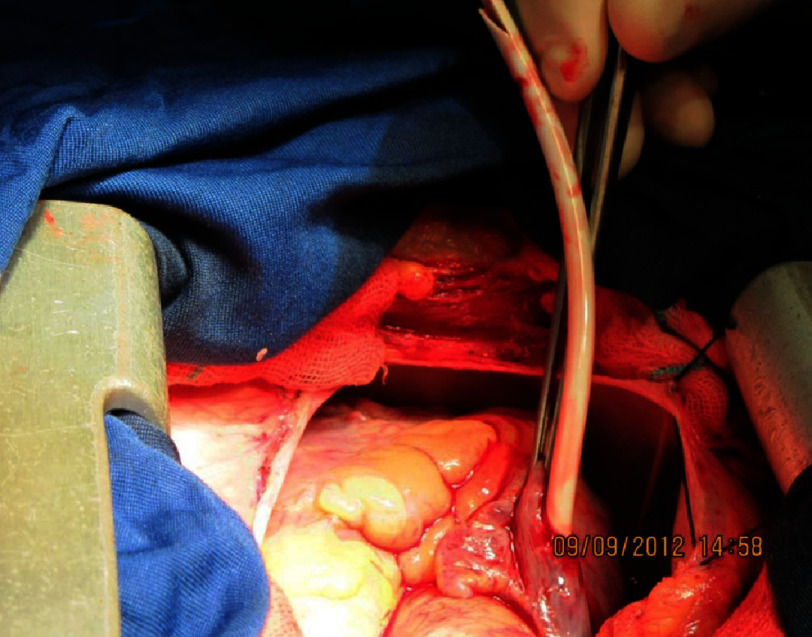
Off-pump retrieval of fractured catheter.

Surgical exploration to remove the catheter remnant was uneventful (Figure 2). The surgery was terminated without blood transfusion. After successful hemostasis and recovery from anesthesia, the patient was transferred to the ICU and extubated. A right internal jugular central line and a radial arterial line were in situ.

## Discussion

Turner & Sommers reported the first case of inadvertent fracture and migration of polyethylene catheter from peripheral vein to right atrium, which in that case ended in fatal outcome^4^. Fisher & Ferreyro evaluated current techniques for nonsurgical removal of intravascular iatrogenic foreign bodies in 220 cases of catheter embolism. They found that unsuccessful removal is associated with a mortality rate of 38%. A piece of fractured catheter may be associated with ruptured of atrial wall and tamponade, myocardial perforation, sepsis, endocarditis, thrombosis, pulmonary embolism, myocardial infarction and arrhythmias^5^.

Shearing or fracture of central venous catheter is a recognized complication. Causes of iatrogenic passage of a fragment of catheter to heart chambers are many and varied. These incidents often result from the introducer needle fracturing the plastic sheath during insertion, bolus infusion causing stress at a fixed catheter tip, or the gradual wear and tear of the catheter’s external layer due to the patient’s movements, leading to its fracture and subsequent migration^6,7^.

Forceable removal of an attached catheter that was previously weakened by frequent and prolonged bending of catheter against of clavicle can cause its fracture and migration. Occasionally the catheter tip is fractured by attaching to the tricuspid valve that caused its gradual weakening and fracture. In one case, the catheter tip migrated across of tricuspid valve and attached to right ventricular wall so that with frequent motion, it ruptured^8^.

Dinkel et al. reported an emergency percutaneous retrieval of a silicone port catheter fragment in the right atrium. Excessive coughing or vigorous sneezing or vomiting, in the presence of catheter fatigue from prolonged use, contributes to in-situ fracture, fragmentation, and/or distal embolization^9^.

The destination site of migrated cathether depends on the angle of cutting edge, sharpness of catheter tip, length, and weight of the catheter. The foreign body can act as a nidus for thrombus formation with resultant pulmonary embolism. Infectious complications include endocarditis, secondary infection of thrombus, mycotic aneurysm, and pulmonary abscesses^10^. In our patient the catheter was retrieved surgically with beating heart surgery without the use of CPB.

## Funding

This research has not received any specific grant from public, commercial, or non-profit sector agencies.

## Conflicts of Interest

The authors have no conflicts of interest to declare.
